# Coupled Effects of Pore Water Velocity and Soil Heterogeneity on Bacterial Transport: Intact vs. Repacked Soils

**DOI:** 10.3389/fmicb.2022.730075

**Published:** 2022-02-21

**Authors:** Jing Chen, Liqiong Yang, Xijuan Chen, Steven Ripp, Jie Zhuang

**Affiliations:** ^1^Key Laboratory of Pollution Ecology and Environmental Engineering, Institute of Applied Ecology, Chinese Academy of Sciences, Shenyang, China; ^2^College of Resources and Environment, University of Chinese Academy of Sciences, Beijing, China; ^3^Center for Environmental Biotechnology, The University of Tennessee, Knoxville, TN, United States; ^4^Department of Biosystems Engineering and Soil Science, The University of Tennessee, Knoxville, TN, United States

**Keywords:** *Escherichia coli*, undisturbed soil, X-ray computed tomography, soil depth, soil structure

## Abstract

Transport of pathogenic bacteria from land surface to groundwater is largely influenced by rainfall intensity and geochemical and structural heterogeneities of subsurface sediments at different depths. It has been assumed that the change in rainfall intensity has different effects on bacterial transport as a function of soil depth. In this study, repacked and intact column systems were used to investigate the influences of pore water velocity on the transport of *Escherichia coli* 652T7 through a loamy soil collected from varying soil depths. The soils differed in geochemical properties and soil structures. The concentrations of bacteria in soil and liquid samples were measured using plate counting method. The breakthrough percentages of *E. coli* 652T7 increased with pore water velocity at each depth in both intact and disturbed soils. Among the different soil depths, the largest velocity effect was observed for the transport through the top soil (0–5 cm) of both disturbed and intact soil profiles. This depth-dependent effect of pore water velocity was attributed to down gradients of soil organic matter (SOM) and iron oxide contents with depth because SOM and iron oxides were favorable for bacterial attachment on soil surfaces. In addition, less bacteria broke through the disturbed soil than through the intact soil at the same depth, and the pore water velocity effect was stronger with the disturbed than intact soils. Specifically, the maximum C/C_0_ (i.e., ratio of effluent to influent concentration) doubled (i.e., from 0.36 to 0.76) in the 0–5 cm intact soil columns and tripled (i.e., from 0.16 to 0.43) in the 0–5 cm repacked soil columns. This structure-dependent effect of pore water velocity was attributed to larger pore tortuosity and a narrower range of pore sizes in the disturbed soil than in the intact soil. These findings suggest that change in pore water velocity could trigger bacterial remobilization especially in surface soils, where more bacteria are retained relative to deep soils.

## Graphical Abstract

Depth profile of pore water velocity effect in differently structured soils.

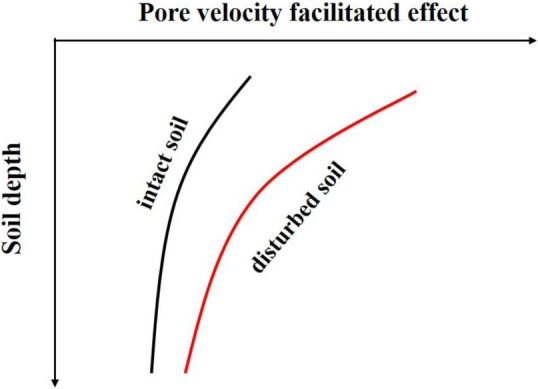

## Introduction

Transport and retention of bacteria in soils are important for understanding and assessing *in situ* bioremediation processes and transport of pathogenic bacteria to groundwater. Bacterial transport can be influenced by soil structure and flow dynamics (Köhne et al., 2009). For instance, the high rainfall intensity increases bacterial release rates from soil by increasing pore water velocity (Blaustein et al., 2016). Rainfall intensity has been considered as a leading factor that influences bacterial release and causes human exposure (Hodgson et al., 2009; Forslund et al., 2011). This problem might become larger as intensive precipitation events become more frequent as a result of climate change (Liu et al., 2013). The bacterial spread could become further significant in soils with macropores, which are favorable to rapid formation of preferential flow. Preferential flow can greatly increase the contamination of water by pathogenic bacteria (Allaire et al., 2009; Li et al., 2017; Guertault and Fox, 2020). Therefore, it is important to understand how soil structure (e.g., macroporosity of intact soil) influence the dependence of bacterial transport on rainfall intensity.

Soil depth represents not only geochemical heterogeneity but also structural heterogeneity in the profiles of subsurface sediments. Geochemical conditions exert significant impacts on bacterial retention and transport in soils and sediments. Previous studies indicated that soil organic matter (SOM) and free iron oxides could enhance the retention of bacteria (Jacobsen et al., 1997; Foppen et al., 2008; Cai et al., 2013; Xu et al., 2019). In addition, soil structure, which determines porosity, pore sizes, and pore connectivity, was reported to influence bacterial transport (Chattopadhyay and Puls, 2000; Vasiliadou and Chrysikopoulos, 2011). Smith et al. (1985) concluded that the transport of bacteria through repacked soil columns was negligible compared to more structured soils. van Elsas et al. (1991) found that a significantly larger amount of *Pseudomonas fluorescens* bacteria moved to deeper soils in intact soil columns than in repacked soil columns. Pore water velocity has been reported to be favorable to bacterial transport (Camper et al., 1993; Bradford et al., 2006; Choi et al., 2007; Syngouna and Chrysikopoulos, 2011; Yang et al., 2021). However, few studies have addressed how the pore water velocity effect varies with soil depth in relation to geochemical and structural gradients. Therefore, the objective of this research was to investigate the depth dependence of the coupled effects of pore water velocity and soil heterogeneities. The results are expected to fill the knowledge gap existing between repacked and intact soil column experiments on bacterial transport.

## Materials and Methods

### Materials

Laboratory transport experiments were conducted with intact and disturbed soils collected from each 5 cm depth of the 0–20 cm topsoil in Shenyang Agro-ecological Research Station, Shenyang, China (123^°^21′E, 41^°^31′N). The soil was a brown earth and classified as fine-loamy, mixed, mesic Typic Hapludalf according to the United States soil taxonomy. The intact cores were collected by vertically driving stainless steel columns (4.5 cm in inner diameter and 5.0 cm in height) into each 5 cm depth of the 0–20 cm field soil. The soil columns were then manually excavated, trimmed and sealed at the two ends with sealing film, and stored under 4^°^C for experimental use. The disturbed columns with the same dimensions of the intact cores were packed with uniformly mixed soil, which was collected from each 5 cm depth, to the same bulk density as that in the corresponding intact cores. Basic physical and chemical properties were analyzed for each 5 cm depth of the 0–20 cm soil. SOM content was determined using the potassium dichromate capacity method-external heating method (Nelson and Sommers, 1996). Free iron oxide content was measured using the dithionite-citrate-bicarbonate (DCB) technique (Mehra and Jackson, 1960).

A gram-negative bacteria strain *E. coli* 652T7 was used as the model bacteria in the transport experiments. It was obtained from the Center for Environmental Biotechnology, University of Tennessee, Knoxville, United States. The cells were grown in Luria Broth medium with a final kanamycin (CAS 133-92-6) concentration of 50 mg L^–1^ at 37°C on a rotary shaker at 160 rpm until a stationary growth phase was reached (∼19.5 h). The cells were harvested by centrifugation (2,000 × g for 20 min) (Universal 320, Hettich, Germany) and then resuspended in NaCl solution (10 mM, pH 6.7) to achieve a target concentration of 2.0 × 10^8^ ( ± 34%) cells mL^–1^.

The X-ray computed tomography (CT) (Phenix Nanotom S, GE, United States) was used to scan the intact and disturbed soil columns at an energy level of 120–140 keV with a pixel size of 22 μm before the transport experiments. The protocol and parameters were detailed in our previous research (Chen et al., 2021). The connectivity plug-in used from BoneJ was designed to estimate the number of connected pores/channels, with connectivity density (CD), total porosity (TP), degree of anisotropy (DA) and specific surface area (SSA). CD is defined as the number of connected pore networks in unit volume of soil core. Higher CD means more intersection of pore channels but less preferential (or macropore) channels. DA means how much orientation there is in the structure. 0.0 means the image is completely isotropic, the sample has no directionality whatsoever. 1.0 means there is an extreme prevailing orientation in the structure of the image.

### Column Experiments

Each experimental setup consisted of the intact/disturbed soil column (Zhongbei Vacuum Equipment Co., Ltd., China), a piston pump (Essentia LC-16, Shimadzu, Japan), and a fraction collector (CF-2, Spectrum Laboratories Inc., Los Angeles, CA, United States). The transport experiments were carried out under steady-state saturated flow conditions at room temperature (25 ± 1°C) in two replicates. The flow rate was adjusted to produce pore water velocities of 6 and 12 cm h^–1^. The column was sealed with an O-ring on each end, and a hydrophilic nylon membrane (30 μm in mesh size) was placed at the top and bottom of the column to prevent possible blockage of the tubing by soil grains during the transport experiments. The columns were saturated by injecting deaerated background solution (10 mM NaCl, pH 6.7) from the bottom for ∼36 h at a low pore water velocity (0.37 cm h^–1^). Then, ∼35 pore volumes of the deaerated background solution was introduced at an experimental pore water velocity (either 6 or 12 cm h^–1^) to standardize the chemical and hydrological conditions of the experimental system. After the effluent stabilized in terms of pH and flow rate, ∼10 pore volumes of the experimental solution (∼8 h at 6 cm h^–1^ and ∼4 h at 12 cm h^–1^), which contained deaerated background solution, bromide (0.3 mM NaBr), and *E. coli* 652T7 (2.0 × 10^8^ ± 34%) cells mL^–1^), were injected into the column at ∼6 or 12 cm h^–1^. During the experiments, a magnetic stirrer was used to uniformly mix the bacterial suspension for stable input into the column. To monitor the stability of bacterial suspension, we measured bacterial concentrations in the input suspension every hour during the entire period of column experiment. The results indicated that the bacterial suspension remained stable. The column effluent was collected every ∼0.3 pore volumes using the fraction collector. The effluent bacterial concentrations were determined by plate counting. Specifically, 0.2 mL of bacteria solution was gradient diluted with 0.9% NaCl solution (i.e., 9 g NaCl in 1,000 mL deionized distilled water). The bacteria and NaCl solution were mixed by a Vortex mixer before 0.2 mL of the mixed solution was spread on a 1.5% LB ager petri dish and incubated at 37°C for 12 h. Although plate counting has some limitations compared to molecular techniques, it can reflect the active bacteria, leading to a better assessment of actual health risk. In addition, plate counting is more accurate than bioluminescence method in the early stage of bacterial breakthrough. Our preliminary experiments with clean quartz sand indicate that the bacterial recovery calculated from plate counting could reach 99 ± 1%, suggesting the feasibility of plate counting. The concentration of the bromide was measured using ion chromatography (ICS-600, Dionex, United States) to estimate the dispersion coefficients (*D*). All soil columns were sterilized at 121°C and 103 kPa for 30 min before the experiments.

### Transport Modeling

The transport process of bacteria was numerically modeled with the classic advection-dispersion equation (ADE) to obtain various mechanistic parameters for quantitative evaluation on the impacts of experimental variables (e.g., pore water velocity, soil depth, and soil structure). Prior to bacterial transport simulation, we used Hydrus-1D software to fit the bromide breakthrough curves to a one-dimensional ADE as follows:


(1) $$\frac{\partial C}{\partial t}=D\frac{\partial^{2}C}{\partial z^{2}}-v\frac{\partial C}{\partial z}$$

In the equation, *C* is bromide concentration (mg L^–1^) or bacterial concentration (N mL^–3^, with N denoting the number of bacteria) in aqueous phase, *t* (h) is time, *D* (cm^2^h^–1^) is dispersion coefficient, *v* (cm h^–1^) is pore water velocity, and *z* (cm) is travel distance (Firouzi et al., 2015). The fitted *D* value was then applied to simulate the bacterial breakthrough curves using a modified ADE form, which includes two-site kinetic attachment-detachment processes. The total bacterial mass balance equation is defined as:


(2) $$\frac{\partial C}{\partial t}+\frac{\rho }{\theta }\frac{\partial S_{1}}{\partial t}+\frac{\rho }{\theta }\frac{\partial S_{2}}{\partial t}=D\frac{\partial^{2}C}{\partial z^{2}}-v\frac{\partial C}{\partial z}$$

(3) $$\frac{\rho }{\theta }\frac{\partial S_{1}}{\partial t}=k_{att1}C-k_{det1}S_{1}\frac{\rho }{\theta }$$

(4) $$\frac{\rho }{\theta }\frac{\partial S_{2}}{\partial t}=k_{att2}C-k_{det2}S_{2}\frac{\rho }{\theta }$$

In the equation, ρ (g cm^–3^) is soil bulk density, θ (cm^3^ cm^–3^) is volumetric water content, *S* (N g^–1^ or cfu kg^–1^) is the bacteria attached on soil surfaces, *k*_*att*_ (h^–1^) is the first-order attachment coefficient, *k*_*det*_ (h^–1^) is the first-order detachment coefficient, and subscripts 1 and 2 refer to the fast and slow kinetic sites, respectively.

## Results

### Dependence of Pore Water Velocity Effect on Soil Depth

**FIGURE 1:**
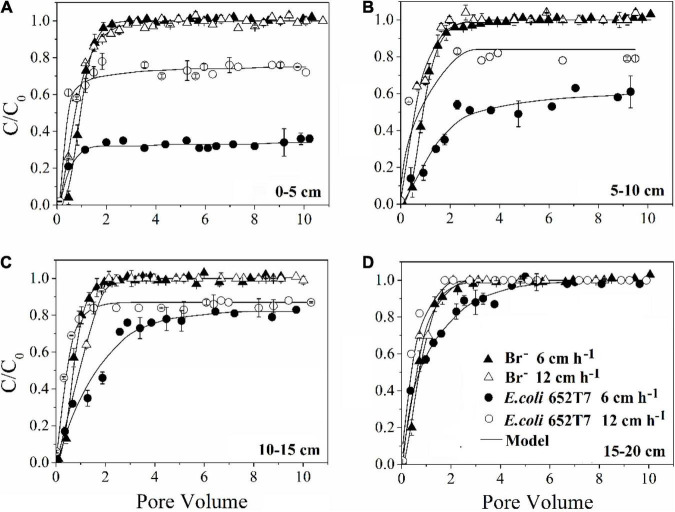
Effect of pore water velocity (6 and 12 cm h^–1^) on the transport of bromide and *E. coli* 652T7 through the intact soils at different depths of **(A)** 0–5 cm, **(B)** 5–10 cm, **(C)** 10–15 cm, and **(D)** 15–20 cm.

The bromide breakthrough curves obtained from different column experiments showed good reproducibility, indicating that the column system was stable and similar under the hydrodynamic conditions (Figure 1).

**FIGURE 2:**
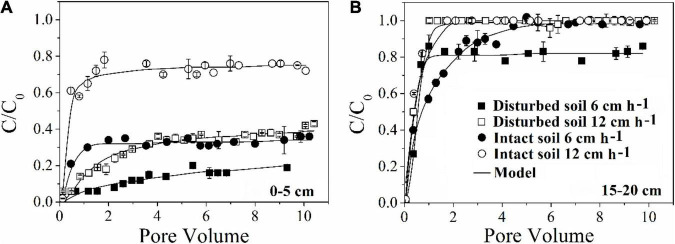
Effect of pore water velocity (6 and 12 cm h^–1^) on the transport of *E. coli* 652T7 through disturbed and intact soil at **(A)** 0–5 cm and **(B)** 15–20 cm.

Our results demonstrated that more bacteria passed through the soil columns at larger pore water velocity. However, the extent of the effect varied with soil depth. In the intact soil columns, the maximum C/C_0_ of bacteria doubled from 0.36 to 0.76 at 0–5 cm depth (Figure 1A) but did not increase at 15–20 cm depth (Figure 1D) when the pore water velocity increased from 6 to 12 cm h^–1^. In the disturbed soil columns, the maximum C/C_0_ nearly tripled from 0.16 to 0.43 at 0–5 cm depth (Figure 2A) and had a small increase (from 0.86 to 1.00) at 15–20 cm depth (Figure 2B). The values of maximum C/C_0_ and *M*_*t*_ indicated that the flow velocity effect decreased with soil depth (Table 1 and Figure 1).

**TABLE 1:** Fitted parameters and mass recovery of bacterial transport experiments.

Soil depth (cm)	Soil structure	*v*	ρ	*D*	*k*_*att*1_	*k*_*att*2_	*k*_*det*1_	*k*_*det*2_	*M*_*t*_
0–5	Disturbed	6.0	1.32	0.15	2,165 ± 10	1,786 ± 10	0.01 ± 0.005	0.85 ± 0.05	13.3 ± 8.0
15–20	Disturbed	6.0	1.28	0.83	135 ± 1	6 ± 0.5	0.09 ± 0.000	23.88 ± 0.10	77.9 ± 0.3
0–5	Disturbed	12.0	1.32	0.59	1,016 ± 1	975 ± 0	0.05 ± 0.002	2.80 ± 0.01	30.2 ± 0.0
15–20	Disturbed	12.0	1.28	2.50	78 ± 12	2 ± 0.1	0.55 ± 0.001	25.00 ± 0.50	95.2 ± 0.1
0–5	Intact	6.0	1.32	0.46	1,488 ± 5	252 ± 5	0.04 ± 0.005	3.00 ± 1.00	31.5 ± 4.7
5–10	Intact	6.0	1.31	0.75	975 ± 5	53 ± 3	0.06 ± 0.005	5.50 ± 0.01	47.4 ± 3.3
10–15	Intact	6.0	1.25	0.80	140 ± 1	9 ± 2	0.08 ± 0.000	20.00 ± 1.00	67.7 ± 8.9
15–20	Intact	6.0	1.28	1.43	120 ± 5	5 ± 0	1.07 ± 0.010	25.00 ± 0.00	86.6 ± 2.5
0–5	Intact	12.0	1.32	1.18	737 ± 3	192 ± 2	0.06 ± 0.005	4.94 ± 0.40	71.0 ± 0.8
5–10	Intact	12.0	1.31	2.85	215 ± 6	19 ± 0	0.07 ± 0.000	105 ± 1.00	75.7 ± 2.3
10–15	Intact	12.0	1.25	4.25	143 ± 5	3 ± 1	0.09 ± 0.002	116 ± 0.00	81.9 ± 1.2
15–20	Intact	12.0	1.28	4.84	6 ± 1	1 ± 0	5.76 ± 0.005	1,973 ± 1.00	96.1 ± 0.9

*v (cm h^–1^) is pore water velocity; ρ (g cm^–3^) is bulk density; D (cm^2^h^–1^) is hydrodynamic dispersion; k_att_ (h^–1^) is first-order attachment coefficient; k_det_ (h^–1^) is first-order detachment coefficient; subscripts 1 and 2 refer to the fast and slow kinetic sites, respectively; M_t_(%) is total percentage of bacterial recovery.*

### Dependence of Pore Water Velocity Effect on Soil Structure

**FIGURE 3:**
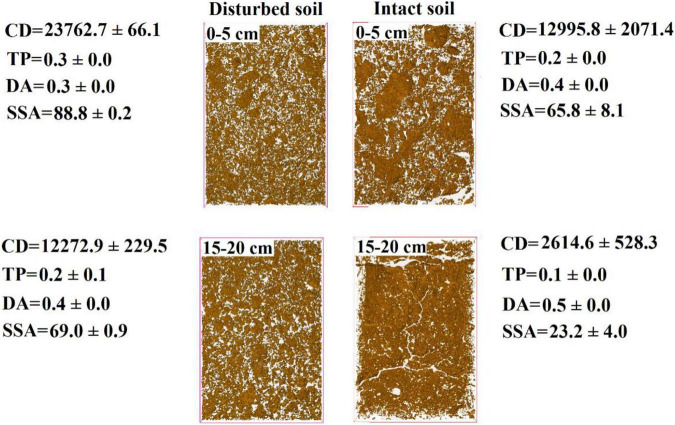
Computed tomography (CT) images of disturbed and intact soil cores at soil depths of 0–5 cm and 15–20 cm. CD is connectivity density of pores (cm^–3^), TP is total porosity (%), DA is degree of anisotropy, SSA is specific surface area (cm^2^ cm^–3^), and the white areas represent soil pore network.

**FIGURE 4:**
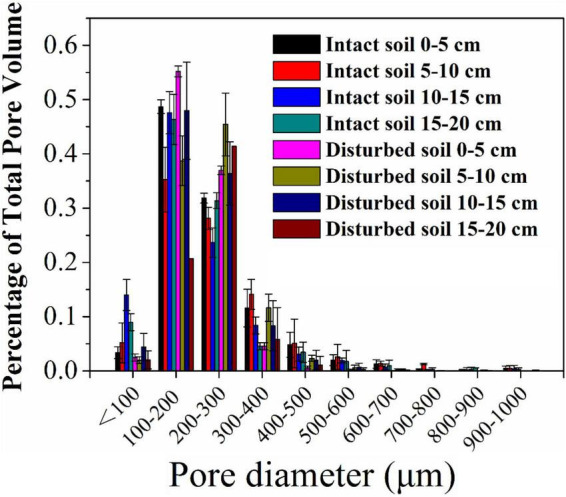
Pore size distribution (% of total pore volume occupied by each pore class) of intact and disturbed soil cores at soil depths of 0–5, 5–10, 10–15, and 15–20 cm.

The pore water velocity effect on bacterial transport was subject to soil structure, but the magnitude of the effect depends on soil geochemistry. This hypothesis was tested by comparing bacterial transport behaviors in the intact and disturbed soils collected from 0–5 to 15–20 cm depths, which represented differences in both soil pore structure and geochemistry. The CT observations showed that the values of pore connectivity density (CD), total porosity (TP), and specific surface area (SSA) were higher and the degree of anisotropy (DA) was lower in the disturbed soils than those in the intact soils (Figure 3). In addition, the range of pore sizes was narrower in the disturbed soil (Figure 4). These results consistently supported the observed velocity effect, which was greater in the disturbed soil than in the intact soil collected from the same soil depth (Figure 2). The maximum C/C_0_ of bacteria increased by 1.7-fold (from 0.16 to 0.43) in the 0–5 cm disturbed soil vs. 1.1-fold (from 0.36 to 0.76) in the 0–5 cm intact soil when the pore water velocity increased from 6 to 12 cm h^–1^. In addition, the higher average pore water velocity resulted in larger values of dispersion coefficients (*D*), and the *D* values were larger in the intact soils than in the disturbed soils at the same pore water velocity. This change suggests that the intact soils had a larger range of pore size distributions than the disturbed soils.

## Discussion

### Dependence of Pore Water Velocity Effect on Soil Surface Chemical Heterogeneity

As indicated by the low values of *k*_*att*_ and high values of *k*_*det*_ in Table 1, bacterial attachment decreased while bacterial detachment increased with pore water velocity. These flow velocity effects decreased with soil depth due to greater retention of the bacteria at 0–5 cm than at 15–20 cm. The discrepancies between bacterial attachment and detachment at different soil depths are partly attributed to soil geochemical heterogeneities (Chen et al., 2021). Table 2 shows that SOM and free iron oxide contents were significantly higher at 0–5 cm than at 15–20 cm. SOM and iron oxides can increase the negative and positive charges on soil surfaces, respectively, influencing bacterial or viral attachment and detachment (Zhuang and Yu, 2002; Zhuang and Jin, 2008). The decrease in bacterial attachment was due to the reduction of contact time and collision efficiency between bacteria and the soil surfaces as the pore water velocity increased (Seuntjens et al., 2001; Pang et al., 2002; Tsang and Lo, 2006; Choi et al., 2007; Johnson et al., 2007). The increase in bacterial detachment was attributed to a loss of balance between the adhesive forces and hydrodynamic forces that acted on bacteria at the solid-water interfaces (SWI) (Bergendahl and Grasso, 2000; Torkzaban et al., 2007).

**TABLE 2:** Basic properties of soils at different depths.

Soil depth (cm)	Organic matter (%)	Free iron oxide (mg kg^–1^)
0–5	3.84 ± 0.03	142.25 ± 1.25
5–10	3.35 ± 0.04	124.87 ± 0.62
10–15	2.86 ± 0.01	115.30 ± 1.59
15–20	2.47 ± 0.04	110.66 ± 1.48

### Dependence of Pore Water Velocity Effect on Pore Structure Heterogeneity

Soil depth would not only cause soil chemical heterogeneity but also change soil structure because SOM and iron oxides can fill soil pores and facilitate soil aggregate formation (McCarthy et al., 2008; Zhuang et al., 2008, 2010; Xu et al., 2020). Therefore, we also attribute the pore water velocity effect to the pore structure heterogeneity, which not only determines bacterial accessibility to various pores but also causes variation of pore water velocity in differently sized pores. Even in the porous media with fairly complex geometries, the local velocity profile is nearly parabolic. The streamlines are faster in the center of the pore channel and slower along the SWI (de Marsily, 1986; Baumann and Werth, 2004; Keller and Auset, 2007). The velocity profile can cause bacterial particles to advect along the central streamlines with fewer detours at a considerably higher rate than those along the rough or tortuous SWI. At the high velocity, the shear forces of flow increase, thus causing an increase in the volume of mobile water in soil pores and shrinkage of stagnant water areas near the soil surface or in the junctions of soil grains. As a result, increase in pore water velocity reduces bacterial dispersion into micropores and bacterial mechanical straining in the contact areas of soil grains (Bradford et al., 2006). Meanwhile, the magnitudes of lift and drag forces acting on bacteria near soil surfaces increase with pore water velocity (Bergendahl and Grasso, 1998; Cushing and Lawler, 1998; Li et al., 2005), facilitating release of the retained bacteria from the soil surfaces (Kuznar and Elimelech, 2007). This theory explained our experimental results well (Figure 2B).

Our CT observations on soil structural heterogeneity indicated a progressive decrease in pore connectivity density (CD) with the depth of intact soils (e.g., from 12,996 ± 2,071 cm^–3^ at 0–5 cm to 2,615 ± 528 cm^–3^ at 15–20 cm) (Figure 3). Since the CD values were positively related to the total numbers of junctions or crossing points of pore channels in unit volume of soil (Doube et al., 2010), the decrease in CD suggests less tortuosity of pore pathways in the deeper soil. Pore tortuosity can increase bacterial travel distance, dispersion in micropores, and collision efficiency against soil surfaces, and thereby bacteria should have higher retention rates in soils with more tortuous pore pathways (e.g., top soil at 0–5 cm) (Chattopadhyay and Puls, 2000; Vasiliadou and Chrysikopoulos, 2011). Our results show that the deeper intact soil had more conductive pores (e.g., straight pore paths), indicating by the increased DA values (from 0.4 ± 0.0 cm^–3^ at 0–5 cm to 0.5 ± 0.0 cm^–3^ at 15–20 cm). Previous study indicated that when the pore water velocity was low, velocity variations among soil pores of different conductivities and sizes were small, and thereby dispersion dominated the bacterial distribution process. When the pore water velocity increased, the variations in velocity became greater among those pores, with increase in velocity in the conductive pores and decrease in velocity in the less conductive pores (Garmeh et al., 2009). Such enlarged difference in the velocity between different pores was more favorable to advective transport of bacteria through the deeper soils relative to the top soils. Considering that bacterial retention in the conductive pores of deeper soils was much less than that in the top soils at lower velocity (e.g., 6 cm h^–1^), the effect of increasing velocity on bacterial transport in deeper soils was thus limited in comparison with that in the top soils. In summary, the pore water velocity effect depends on bacterial accessibility and distribution in different sizes of pores, with the effect being larger in top soil or in the pores where bacterial abundance is larger.

To verify the dependence of pore water velocity effect on pore structure heterogeneity, we compared bacterial transport behaviors in the intact soil columns with the repacked soil columns. The two sets of soil columns had the same soil geochemical and textural conditions but different soil structure due to soil disturbance during the repacking process. The CD values of the disturbed soils (23,763 ± 66 cm^–3^ at 0–5 cm and 12,273 ± 229 cm^–3^ at 15–20 cm) approximately doubled those of the intact soils at the same depths (12,996 ± 2,071 cm^–3^ at 0–5 cm and 2,615 ± 528 cm^–3^ at 15–20 cm). The pore pathways were more tortuous in the disturbed soil than in the intact soils, leading to more retention of the bacteria in the disturbed soils due likely to mechanical straining. As a result, a portion of the injected bacteria, which would otherwise be retained at a lower velocity (e.g., 6 cm h^–1^), were mobilized at a higher velocity (e.g., 12 cm h^–1^). Our CT observation also showed that the range of pore sizes was narrower in the disturbed soil (Figure 4), resulting in less variation of pore water velocity in comparison with the larger variation in the intact soils, which had a wider range of pore sizes due to the existence of conductive larger pores. As a result, the increase in pore water velocity (or pump rate) resulted in faster transport of the bacteria through larger pores and slower movement in smaller pores (Smith et al., 1985). However, if bacteria are able to transport fast in the conductive pore network (e.g., macropores) at a lower pore water velocity, doubling it might not significantly increase the transport of bacteria (Martins et al., 2013). Therefore, the improvement of the overall flow velocity effect was more significantly determined by the bacterial abundance in the less conductive, smaller pores, which dominated bacterial retention in the disturbed soils.

## Conclusion

This study demonstrated that bacterial transport through soils increased with pore water velocity and that the effect decreased with soil depth or down-gradients of SOM and iron oxide contents. The pore water velocity effect was caused by increases in hydrodynamic forces and bacterial dispersion into micropores. The pore water velocity effect was smaller in soils with less SOM and iron oxides. As a result, the bacterial transport facilitated by pore water velocity became smaller in deeper soils. In addition, the pore water velocity effect was greater in disturbed soil than in intact soil at the same depth or under the same geochemical conditions. This structural effect was resulted from soil repacking, which increased pore tortuosity and path length of bacterial travel. Overall, the effect of pore water velocity depends on bacterial attachment on soil surfaces. The stronger or more they are retained on soil, the larger the pore water velocity effect is. This study provides an important scientific foundation for accurate assessment of the breakthrough of pathogenic bacteria from geochemically and structurally heterogeneous soil profiles. Also, the results imply that a soil depth at which change in pore water velocity disappears under natural precipitation or irrigation conditions might be the maximum depth for preventing pathogen spread and protecting groundwater from pathogenic bacterial contamination. The size of bacteria in natural soil may be smaller and affected by protozoan. This study only represents a portion of bacteria, and the effect of pore water velocity on smaller bacteria needs to be studied in the future.

## Data Availability Statement

The original contributions presented in the study are included in the article/supplementary material, further inquiries can be directed to the corresponding author/s.

## Author Contributions

JC performed the experiments. LY analyzed the data and wrote the manuscript. JZ designed the experiments. XC, SR, and JZ reviewed the manuscript. All authors contributed to the article and approved the submitted version.

## Conflict of Interest

The authors declare that the research was conducted in the absence of any commercial or financial relationships that could be construed as a potential conflict of interest.

## Publisher’s Note

All claims expressed in this article are solely those of the authors and do not necessarily represent those of their affiliated organizations, or those of the publisher, the editors and the reviewers. Any product that may be evaluated in this article, or claim that may be made by its manufacturer, is not guaranteed or endorsed by the publisher.
